# Clinical and radiographic outcomes of a trabecular titanium™ acetabular component in hip arthroplasty: results at minimum 5 years follow-up

**DOI:** 10.1186/s12891-015-0822-9

**Published:** 2015-12-03

**Authors:** Loris Perticarini, Giacomo Zanon, Stefano Marco Paolo Rossi, Francesco M. Benazzo

**Affiliations:** Clinica Ortopedica e Traumatologica, Università degli Studi di Pavia, Fondazione IRCCS Policlinico San Matteo, Pavia, Viale Camillo Golgi 19, 27100 Pavia, Italy

## Abstract

**Background:**

Aim of this prospective study was to evaluate mid-term clinical and radiographic outcomes in total hip arthroplasty using an acetabular cup made of an innovative biomaterial, Trabecular Titanium™, whose highly porous structure and mechanical properties have been designed to mimic those of the natural bone, thus promoting a more physiological load transfer and a more durable fixation.

**Methods:**

Between September 2007 and November 2009, 134 total hip replacements and eight revisions were carried out using DELTA-TT primary cups (Lima Corporate, Villanova di San Daniele del Friuli, Italy) in 133 consecutive patients. Mean age was 57.5 ± 14.7 SD (18–92) years. Diagnosis was primarily hip osteoarthritis in 85 (63 %) cases, developmental dysplasia of the hip (DDH) in 24 (18 %) and hip avascular necrosis (AVN) in 10 (7 %). All the revision procedures were due to aseptic loosening of the original implant. Approval of the Institutional Review Board of the IRCCS Policlinico San Matteo in Pavia was obtained for this study.

**Results:**

Mean follow-up was 72.7 ± 7.9 SD (60–86) months. Average Harris Hip Score (HHS) significantly increased from 44.2 ± 5.4 SD (35–52) preoperatively to 95.9 ± 3.5 SD (88–100) at the last follow-up. No major post-operative complications were observed. 99.3 % of the acetabular components were radiographically stable at the last follow-up, without any radiolucent lines, sclerotic areas or periprosthetic osteolysis. Kaplan-Meier survival rate was 99.3 % at 5 years (95 % confidence interval).

**Conclusions:**

This first account on the mid-term clinical performance of the DELTA-TT cup shows primary and secondary stability, thus representing an optimal solution for patients with high demands or affected by severe hip conditions.

## Background

Total hip arthroplasty (THA) is currently one of the most widely performed procedures in orthopaedic practice, proving remarkably successful in providing pain relief and restoring joint function. THA has been continuously evolving in the past years in terms of prosthetic designs and materials, surgical techniques, treatment and prevention of complications and patient postoperative management. Although major improvements have been recorded with regard to clinical outcomes and survivorship, acetabular component loosening remains among the most common causes of failure and revision [1–4]. Patient age, poor bone quality and conditions, such as osteonecrosis and dysplasia, have been observed to influence negatively long-term clinical results [5–8].

Prerequisites to achieve durable cementless cup fixation are close contact with viable native bone, primary mechanical stability and secondary bone integration. Press-fit techniques, bone-implant apposition, pore size and material properties play a major role in promoting bone ingrowth and long-term fixation [2–4]. Trabecular Titanium™ is a highly porous cellular solid structure, designed with multi-planar hexagonal interconnected cells to mimic the trabecular morphology of the natural bone [9, 10]. The continuity between the porous and solid parts has been specifically developed to overcome the limitations of the traditional porous coatings. In fact, the absence of an interface between the trabecular structure and the bulk material provides greater structural solidity and thus higher resistance to detachment and corrosion [11]. Trabecular Titanium™ has also shown osteoinductive and osteconductive properties in various in vitro and animal studies, where it has demonstrated to stimulate vascularisation as well as osteoblast proliferation and differentiation [12–15].

**Fig. 1:**
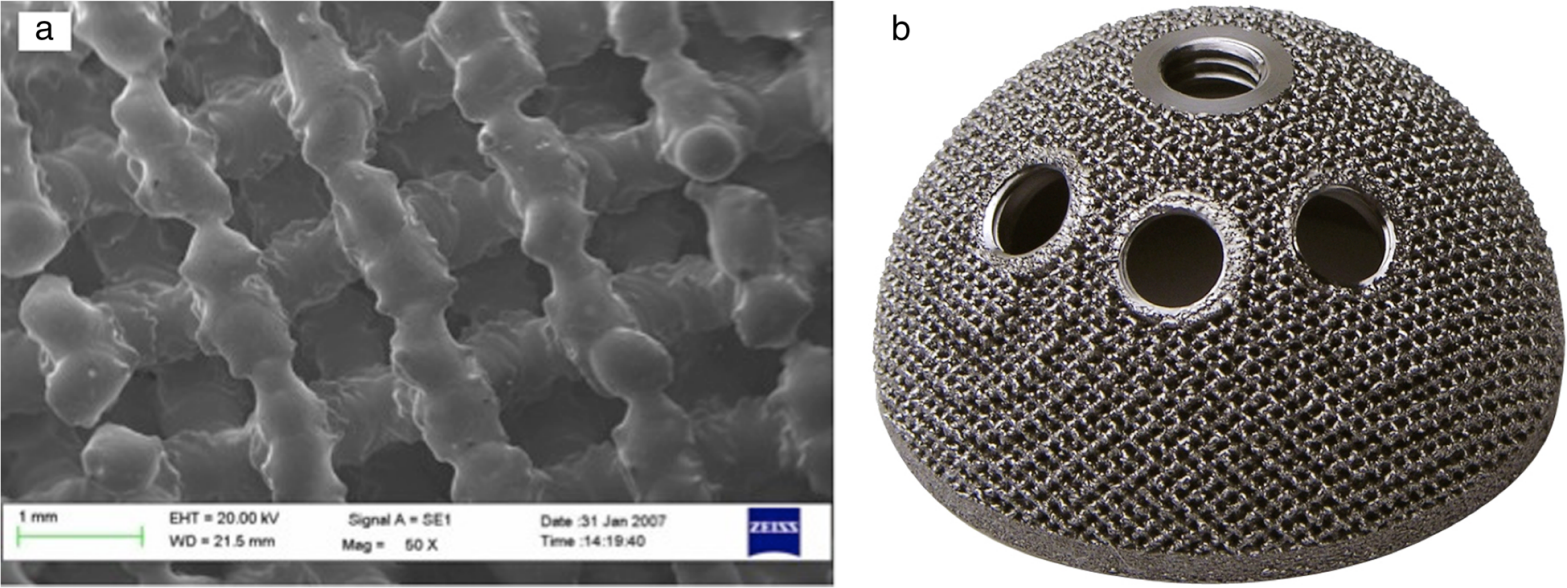
**a ** SEM image of the Trabecular Titanium cellular solid structure; **b** Cementless acetabular cup in Trabecular Titanium™ (DELTA-TT, Lima Corporate)

Aim of this prospective study was to evaluate mid-term clinical and radiographic outcomes of a Trabecular Titanium™ cup (Fig. 1) in difficult THA cases, such as patients with high demands, subjects affected by severe hip conditions (*i.e.* osteonecrosis, dysplasia) or with extremely poor bone quality.

## Methods

### Patient population

Between September 2007 and November 2009, 134 hip replacements and eight revisions were carried out using DELTA-TT primary acetabular cups in 133 consecutive patients, 59 (44 %) men, 74 (56 %) women.

The acetabular component was implanted at the right side in 76 cases and at the left side in 66 cases in this prospective case series. Three women underwent a bilateral single-stage procedure, while two women and four men were operated on the other side averagely 11 months after the first surgery. The mean age of patients was 57.5 ± 14.7 SD (18–92) years (Table 1).

**Table 1:** Patients characteristics in terms of age (years), weight (kg), height (cm) and BMI (kg/cm^2^)

	Age (years)	Weight (kg)	Height (cm)	BMI (kg/cm^2^).
Mean	57,5	71,4	166	25,8
Min	16	45	148	18,7
Max	92	100	185	39,6

The patients were selected from a population requiring total hip arthroplasty (THA) according to the following eligibility criteria: males and females, with a BMI ≤40 kg/m^2^, presenting pain, limp and severe functional impairment. The patients needed to be assessed as difficult THA cases in order to be included in this series, such as subjects affected by severe hip conditions (i.e. osteonecrosis, dysplasia), patients with high demands or extremely poor bone quality according to the patient’s age and X-ray evaluation, or patients requiring hip revision surgery but presenting limited bone deficiency (i.e. Paprosky I or IIA). The following pathologies were regarded as contraindications for inclusion in this investigation: severe and impairing vascular diseases, neurosensory deficits, neuromuscular diseases, tumours, active or suspicious infections, severe kidney failure, Paget’s disease. Underlying pathology was hip osteoarthritis (OA) in 85 (63 %) cases, developmental dysplasia of the hip (DDH) in 24 (18 %), hip avascular necrosis (AVN) in 10 (7 %), sub-capital hip fractures in 7 (5 %), post-traumatic arthritis in 5 (4 %) (1 case of lag screw cut-out of femoral nail), sequelae of femoral varus osteotomy in hip dysplasia in 1 (1 %), deformity following epiphysiolysis of the femoral head in 1 hip (1 %), and rheumatoid arthritis in 1 (1 %). All the revision procedures were due to aseptic loosening of the original implant, and the patients presented bone defects classified as Paprosky Type I or IIA.

Approval of the Institutional Review Board of the IRCCS Policlinico San Matteo in Pavia was obtained for this study, and all subjects provided informed consent prior to participation.

### Acetabular and femoral components

Uncemented DELTA-TT cups (Lima Corporate, Villanova di San Daniele del Friuli, Italy) were used in all cases (Fig. 1). Their extremely rough surface is specifically designed to enhance the mechanical grip and ensure maximum stability, while promoting an effective bone integration. The shell of this cup is manufactured in titanium alloy (Ti_6_Al_4_V) via Electron Beam Melting [9, 10]. This one-step production process guarantees structural continuity between the inner solid part and the external porous surface, reducing the typical risks of prosthetic components with macro-rough coatings, such as galvanic effects and detachment [11]. Tension tests performed on Trabecular Titanium™ showed that its tensile strength (i.e. 86.4 MPa) exceeds that of most current coatings and porous materials [9]. The structure of Trabecular Titanium™ is characterized by an average porosity of 65 %, with a mean pore diameter of 640 *μ*m [9].

Cup diameters ranged from 44 to 60 mm. Hip reinforcement screws were used in 17 cases, as a further safety measure in presence of poor bone quality (severe osteoporosis), in DDH patients and in some difficult cases of sub-capital fracture.

Bearing couplings were ceramic-on-ceramic in 131 patients (92 %), and polyethylene-on-ceramic in the remaining cases. Polyethylene liners were inserted with their long 10° lip in the best position after a trial reduction.

All the femoral heads were made of *BIOLOX*® Delta ceramic (CeramTec GmbH, Plochingen, Germany), and their size ranged from 28 to 40 mm.

Cups were associated to straight stems (C2 stem [Lima Corporate] in 38 cases, lateralizing C2 stem [Lima Corporate] in 45 cases, Zweymüller stem in two cases) and to modular conical stems (Modulus and Revision stems [Lima Corporate] in 51 and four cases, respectively) to restore the correct hip offset and leg length.

### Surgical procedure

A postero-lateral approach with lateral decubitus position was used in all cases. The acetabulum was up-reamed by 1 mm (line-to-line fixation) to accommodate for the surface interference of the DELTA-TT cup. The acetabular component was impacted with abduction and anteversion angles adapted to that of the patient’s anatomy, or corrected according to the need in dysplastic cases.

### Clinical and radiographic assessments

Clinical evaluation with the Harris Hip Score (HHS) [16] was performed preoperatively, at 3, 6, 12, months, and then annually thereafter. At the same time, radiographic analysis was carried out to measure the cup inclination angle and to identify the presence of any radiolucent lines, osteolysis and sclerosis, according to the three zones defined by DeLee and Charnley [17].

Radiographic examination included views, such as antero-posterior (AP) of the pelvis and lateral of the affected hip, according to a standard protocol for imaging reproducibility. A single observer made all measurements.

Patients were monitored in order to check for intra- and post-operative complications. A Kaplan-Meier analysis was used to estimate survivorship using acetabular revision due to loosening as the end-point.

## Results

### Clinical evaluation

Mean follow-up was 72.7 ± 7.9 SD (60–86) months. Average total HHS improved remarkably, from 44.2 ± 5.4 SD (35–52) preoperatively to 95.9 ± 3.5 SD (88–100) at the last follow-up (Table 2). No differences were observed in terms of clinical results and survivorship among the three major diagnoses (*i.e.* OA, AVN and DDH). Significant functional recovery and pain relief were recorded in all cases. All patients return to routine activities of daily living with no limitations, showing good mobility and joint functionality.

**Table 2:** Time pattern (months, m) of Harris Hip Score (HHS) at 5 years follow-up

HHS	Pre-op	1 m	3 m	6 m	12 m	24 m	36 m	48 m	60 m
Mean	44,2	87,5	94,1	96,1	96,7	96,0	95,5	95,9	95,9
Min	35	80	70	85	80	88	87	88	88
Max	52	93	100	100	100	100	100	100	100

No major complications were observed. Hip dislocation occurred postoperatively in two patients but they were treated conservatively. One case of pericarditis occurred 1 month after surgery, and was treated with antibiotic prophylaxis for 6 months.

Two cases of squeaking were reported. The first one was solved with three local injections of low molecular weight hyaluronic acid under image intensifier, while the second one underwent spontaneous resolution.

One death unrelated to the surgical procedure occurred 2 months after a contralateral THA was performed. All the remaining patients completed the clinical and radiographic evaluation.

### Radiographic evaluation

**Fig. 2:**
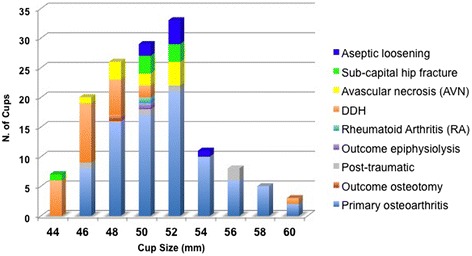
Distribution of cup size (mm) according to primary diagnosis

Postoperatively, the mean cup abduction angle was 41.7° ± 6.2 SD (28°–54°). 99.3 % of the acetabular components were radiographically stable at the last follow-up, showing evident signs of bone remodelling and integration [18], without any radiolucent lines, sclerotic areas or periprosthetic osteolysis (Fig. 2).

### Survivorship

**Fig. 3:**
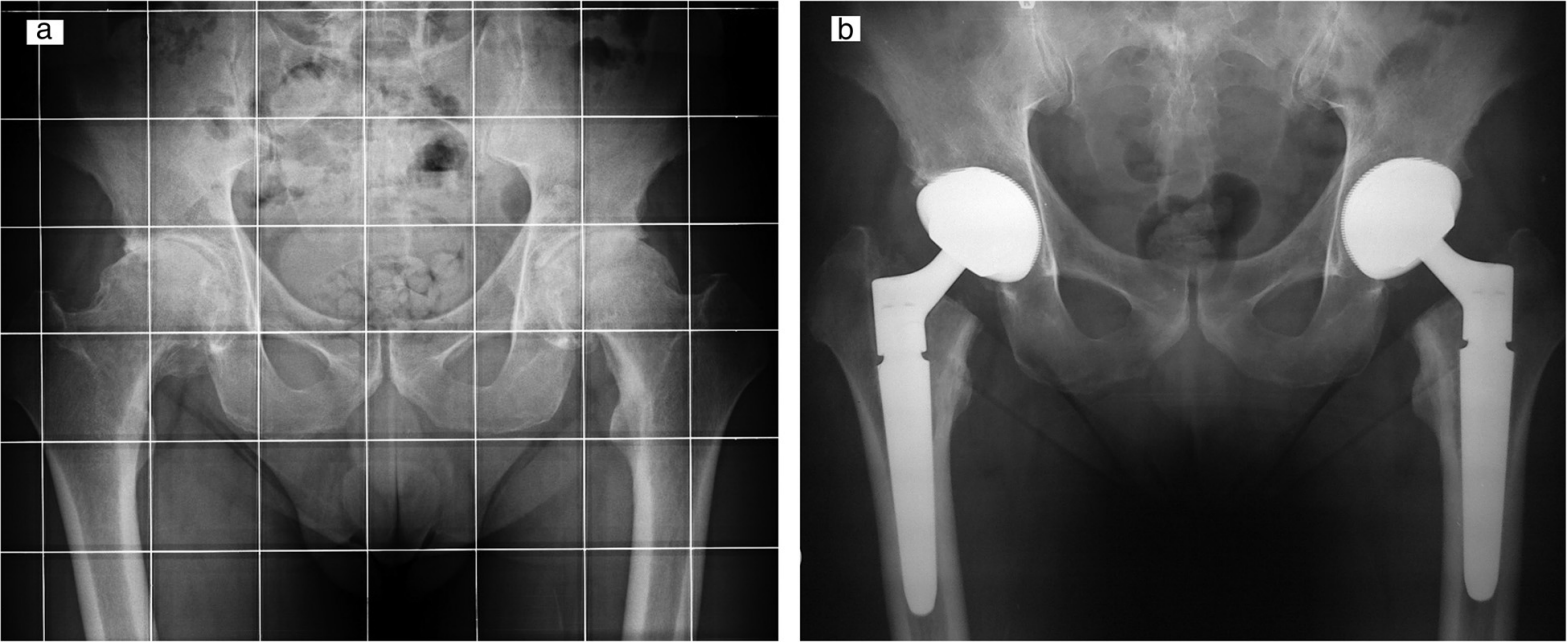
Bilateral hip osteoarthritis in young patient: X-ray pre-operatory (**a**) and at 3-year follow-up (**b**)

**Fig. 4:**
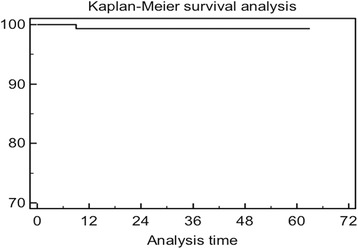
Implant survivorship according to Kaplan-Meier analysis, using revision of the acetabular component for loosening as the endpoint

**Fig. 5:**
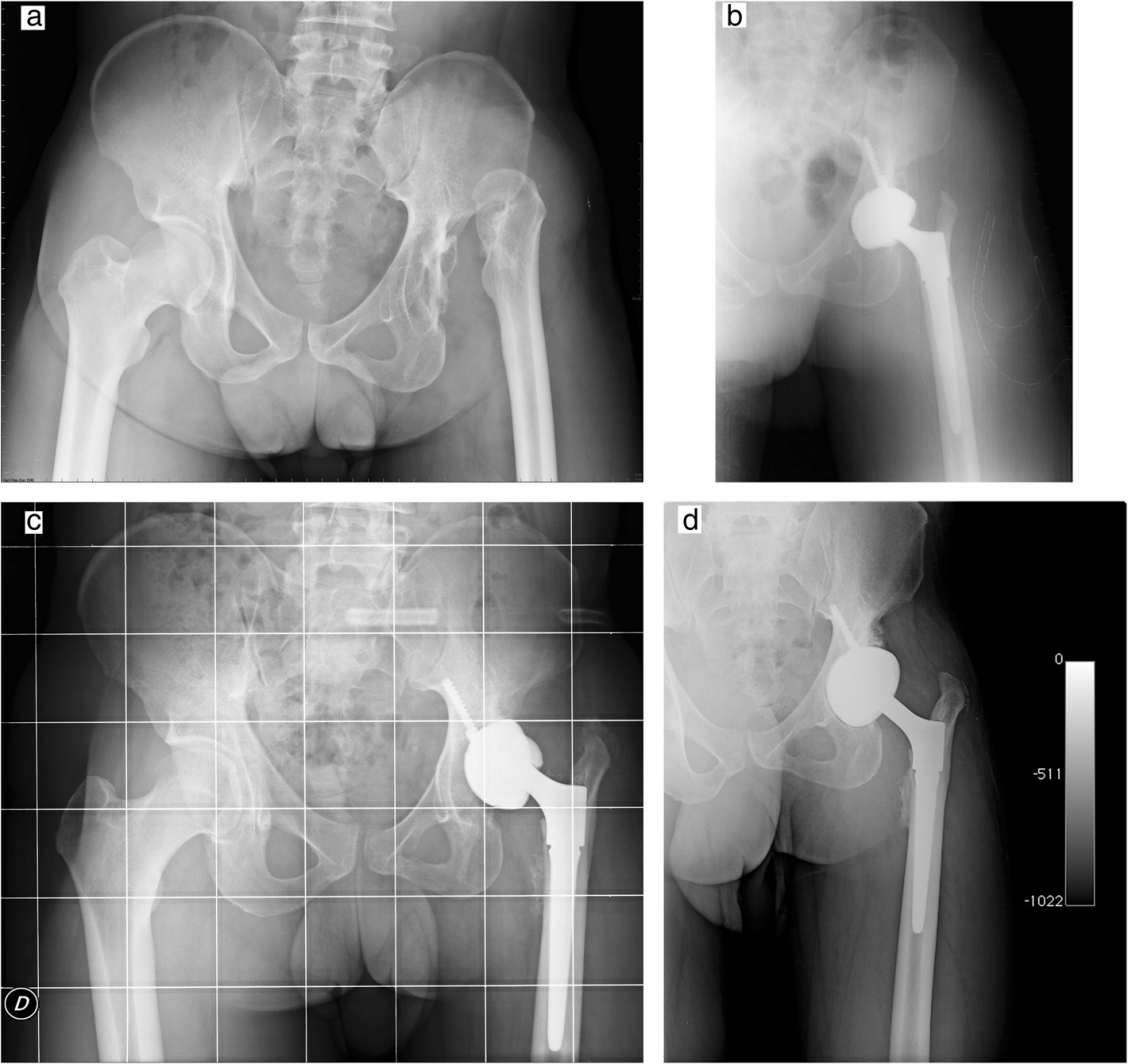
Case of cup loosening: **a** pre-operative X-ray (DDH Crowe 4), **b** post-operative radiograph, **c** loosening of the cup, **d** X-ray at 4-year follow-up, after revision with primary DELTA-TT cup

Kaplan-Meier cumulative survivorship was 99.5 % (95 % confidence interval) at 5 years (Fig. 3). One case of aseptic loosening occurred in a Crowe 4 DDH patient 9 months after surgery, due to the high dislodging forces of the lengthened abductor muscles (no femoral shortening was performed). This patient underwent a revision with another DELTA-TT cup, with excellent results at 5-year follow-up (Figs. 4, 5).

Two patients underwent a stem revision for thigh pain, respectively 11 and 42 months after surgery, with complete resolution of symptoms.

## Discussion

Multiples studies report short- to mid-term clinical outcomes with a large variety of cementless acetabular cups [1–4, 19–22]. Early results with Trabecular Metal (TM, Zimmer) cups reported excellent stability and bone apposition, both in primary and revision cases, at a mean follow-up of 28 months; only one patient out of 62 resulted unsatisfied due to persistent thigh pain [19]. Kamada et al. [20] assessed the log-term clinical and radiographic outcomes of Trabecular Metal cups on 45 cases of hip dysplasia. At a mean follow-up of 9.8 years (range 7–10 years), all cups resulted radiographically stable, with a mean JOA hip score improving from 48 to 92 points. Naziri et al. [21] reported no radiolucencies or cup migration on 252 patients using a highly porous titanium cup at a minimum follow-up of 3 years. Naudie et al. [22] performed a radiostereometric analysis on an acetabular shell with an enhanced porous ingrowth surface, describing minimal cup micromotions at short-term follow-up. Nonetheless, most of the current acetabular coatings and highly porous cup designs have shown to present inherent limitations, such as low volumetric porosity, relatively high modulus of elasticity and low frictional properties [1–4, 19–22].

This study reports a first account on the mid-term clinical performance of the DELTA-TT cup, with a survivorship of 99.3 % at 5 years. Excellent short- and mid-term radiographic and clinical results were observed, with a significant increase in patients’ quality of life and joint functional status, both in primary and revision cases.

No differences were observed in terms of clinical outcomes according to age or different primary diagnoses, such osteonecrosis and dysplasia. Age distribution in the patient population deliberately encompassed subjects with high demands as well as elderly, in order to be able to observe and capitalize on all the features of this acetabular system, such as its high grip and osseointegration potential.

All cups showed excellent primary stability and good osseointegration, even in patients with poor bone quality. The tight “scratch-fit” of this cup has exhibited a superior initial fixation, while reducing the risk of micromotions. Trabecular Titanium™ is in fact characterized by an extremely high friction coefficient thanks to its macro-porous structure which fosters the mechanical interlocking at the bone-implant interface.

99.3 % of the acetabular components resulted radiographic stable, without radiolucent lines or periprosthetic osteolysis. DELTA-TT cups have been designed to promote a more physiological load transfer, hence minimizing the potential for stress shielding and bone resorption. In virtue of its highly porous hexagonal cell structure, Trabecular Titanium™ displays in fact an elastic modulus (1.1 GPA) [9] largely inferior to the other materials used in the orthopaedic field, but comparable to the one of the trabecular bone.

Ceramic-on-ceramic bearings and large diameter heads were mostly used during the course of this investigation to increase ROM and minimize wear, in order to reduce the incidence of potential revision at long-term.

## Conclusions

In our experience, these clinical results obtained with a patient population characterized by a wide range of age suggest that DELTA-TT cups can provide an optimal prosthetic solution both in high-demand patients, with ceramic-on-ceramic bearings, as well as in elderly patients, with poor bone quality, using preferentially ceramic-on-polyethylene. Further studies are necessary to assess long-term survivorship.

## Abbreviations

AVN: avascular necrosis
BMI: body mass index
DDH: developmental dysplasia of the hip
HHS: Harris Hip Score
OA: osteoarthritis
SEM: scanning electron microscopy
TT: Trabecular Titanium
